# Dermatillomania: A Case Report and Literature Review

**DOI:** 10.7759/cureus.12932

**Published:** 2021-01-27

**Authors:** Srikrishna V Malayala, Hira Rehman, Deepa Vasireddy

**Affiliations:** 1 Internal Medicine, Temple University Hospital, Philadelphia, USA; 2 Internal Medicine, Physicians for American Healthcare Access, Philadelphia, USA; 3 Pediatrics, Pediatric Group of Acadiana, Lafayette, USA

**Keywords:** ocd/ anxiety disorders, wound infections, obsessive-compulsive symptoms, skin lesions, pruritis, dermatillomania, skin picking, ssri

## Abstract

Skin picking disorder, also termed dermatillomania is a condition that leads to repetitive picking of their skin ending up in skin and soft tissue damage. It is classified in Diagnostic and Statistical Manual of Mental Disorder Fifth edition under the "obsessive compulsive and related disorders" section. Often associated with other psychiatric conditions like autism, alcohol abuse, obsessive compulsive, body dysmorphic, mood, anxiety and borderline personality disorders, it is a disorder that is quite often underreported. The patient in this case report is a 58-year-old male with a diagnosis of obsessive compulsive disorder (OCD) who reported severe anxiety and skin picking episodes over several years. He presented to the emergency room with an extensive wound on distal left foot with exposure of the underlying muscle tissue, that resulted from the excessive picking of skin from the left foot. This compulsive behavior started off with picking the skin around his nail beds and slowly got worse. The skin picking would get worse whenever he gets nervous or anxious. The wound was treated with topical wound care and antibiotics. At the time of discharge, he was prescribed oral antibiotics to complete his course of treatment and was referred to the hospital's cognitive behavioral therapy (CBT) program that specializes in treatment of OCD and anxiety disorders. Treatment of dermatillomania is a multipronged approach and should include treatment of the underlying psychiatric illness, the treatment for pruritus and topical treatment of the lesions. Selective serotonin reuptake inhibitors (SSRIs) have proved to be the most effective in treating the psychiatric component of dermatillomania. Non-pharmacological treatments such as behavioral therapy, habit reversal exercises and support groups have also proved to be helpful and are well tolerated amongst patients suffering from dermatillomania.

## Introduction

Dermatillomania also known as skin picking disorder causes those affected, to repetitively pick at their skin leading to soft tissue damage. It is a disorder that is believed to be underreported and the prevalence in the general population remains unknown [1,2]. American Psychiatric Association's (APA) “Diagnostic and Statistical Manual of Mental Disorder” Fifth edition (DSM-5) has classified dermatillomania under obsessive compulsive and related disorders. The criteria for the disorder per DSM-5 are:

(a) recurrent skin picking resulting in skin lesions;

(b) repeated attempts to decrease or stop skin picking;

(c) the skin picking causes clinically significant distress or impairment in social, occupational, or other important areas of functioning;

(d) the skin picking is not attributable to the physiological effects of a substance or another medical condition; and

(e) the skin picking is not better explained by symptoms of another mental disorder [3].

The age of onset may be during childhood, adolescence or adulthood but generally it tends to be in adolescence between ages 13 and 15 years of age [4,5]. Several psychiatric co-morbidities have been found to be associated with skin picking leading to self-injury such as obsessive compulsive disorder (OCD), alcohol abuse or dependence, body dysmorphic disorder, mood disorder, anxiety disorder, borderline personality disorder and obsessive compulsive personality disorder [6]. Certain syndromes such as Prader- Willi syndrome, Lesch-Nyhan syndrome, Smith-Magenis syndrome and Tourette syndrome are also reported to be associated [7-10]. Individuals with autism have been observed to exhibit skin picking as well [11].

With this case report, we present a case of a severe case of dermatillomania requiring hospitalization for the wound that the patient induced onto himself. Our objective with this case report is to present the case and summarize the available and existing literature about the "skin picking disorder" also known as dermatillomania. 

## Case presentation

The patient is a 58-year-old male residing in the city of Philadelphia, Pennsylvania, USA. He lives by himself in an apartment. He is currently disabled but was working at an appliance repair store up until a few years ago. He carries a history of hypertension, type 2 diabetes mellitus, hypertriglyceridemia and lumbar spondylosis. He is an active smoker and smokes approximately a pack of cigarettes daily. He reported compliance to all his medications (25 mg hydrochlorothiazide and 80 mg valsartan that he takes every day for hypertension; 500 mg twice daily of metformin and 50 mg daily sitagliptin for type 2 diabetes mellitus; 20 mg daily pravastatin and over the counter fish oil for the hypertriglyceridemia). Other than smoking, he denied any concern in terms of the complications from these comorbidities. Urine toxicology screen was negative for any illicit substances. He denied peripheral vascular disease (PVD), cardiovascular or neurological conditions though he did not recall getting tested for PVD or diabetic neuropathy as such.

He was also diagnosed with OCD at the age of 26 years but reported compulsive behavior from his adolescence. As an adolescent, he first developed compulsive squeezing of his acne and picking of his facial hair. A few years later, he had to compulsively pick the hair, the acne and skin in his entire body, more so in his legs. At times, he reported severe anxiety associated with these skin-picking episodes and even reported a couple of visits to the emergency room with the symptoms of OCD. He was taking quetiapine 50 mg daily for his OCD. 

Over the last few months or so, he reported he became more and more compulsive in picking the skin on his toes and his feet. Though he was not able to specify the exact times he was picking his skin every day, he did report that the picking would get worse whenever he gets nervous and anxious. He described his picking as constant on a daily basis, sometimes that episode could last for minutes. This compulsive behavior started off with picking the skin around his nail beds and slowly got worse. He reported few occasions of having painful sores and wounds due to the skin picking when he applied topical over the counter antibiotic and antiseptic creams. However, he could not resist picking the scars over these wounds and the wounds would never heal. Over time, he reported that “he gave up” on his skin picking behavior as he noticed that the left leg wound was gradually getting worse. Due to the worsening pain and foul-smelling discharge from the wound, he decided to come to the emergency room (ER).

**Figure 1:**
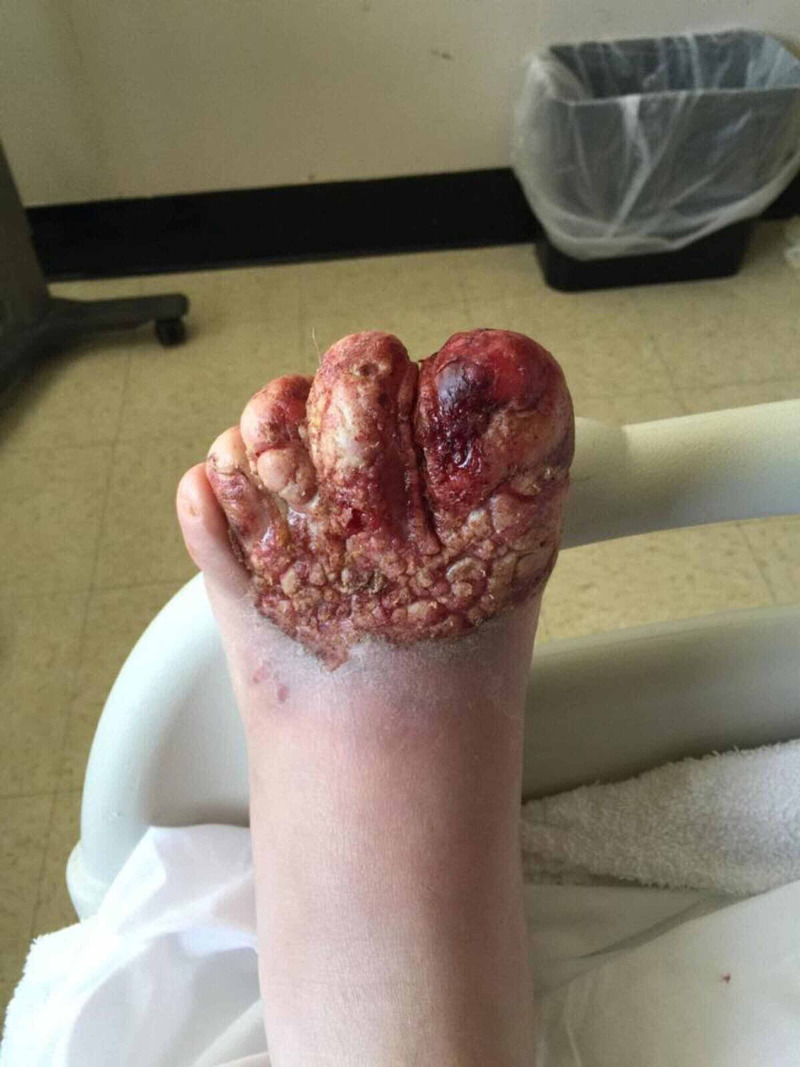
Dermatillomania leading to infected left distal foot wound. Patient in this case presented with an extensive wound on distal left foot with exposure of the underlying muscle tissue, oozing of blood and surrounding erythema. This was treated with topical wound care and broad-spectrum antibiotics. He was further treated with cognitive behavioral therapy and atypical antipsychotics.

When he presented to the ER, he was found to have an extensive wound on distal left foot with exposure of the underlying muscle tissue, oozing of blood and surrounding erythema (Figure 1).

He had a low-grade temperature of 100.2 F, heart rate of 103 beats/min and blood pressure 133/82 mm Hg. Systemic examination was normal other than sinus tachycardia and the above-described wound. The patient’s initial baseline laboratory data at the time of admission showed a leukocytosis with a white cell count up to 13,400 per microliter. The patient’s thyroid, renal, electrolyte, acid base and liver function tests were within normal limits. He was referred to admission to the general medicine service along with broad-spectrum intravenous (IV) antibiotic coverage (vancomycin and piperacillin/tazobactam) to empirically treat methicillin-resistant Staphylococcus aureus (MRSA), other gram-positive, gram-negative and anaerobic organisms that might be causing the wound infection. The IV antibiotics were given for about 72 hours till the wound cultures were finalized and the blood cultures resulted negative. The wound was also evaluated by general surgery service who recommend topical wound care and management with antibiotics.

With the intention to treat the underlying disease, we investigated his psychiatric history in detail. He was under the care of a few psychiatrists for a diagnosis of OCD over the last 30 years or so. He was on a variety of different medications for his OCD including aripiprazole, risperidone and fluoxetine. He did not report a great benefit from any of these medications. Apart from the history of smoking, he denied alcohol or substance abuse. He denied any suicidal attempts. He noticed that his skin picking would get worse whenever he gets nervous or anxious. He reported that his mother and sister also had similar obsessive compulsive traits. When he started realizing that his symptoms were getting worse of late and when the medications did not seem to help, he decided to join a few online support groups on the internet. He felt relieved talking to others with similar symptoms and dedicated some time every day talking and listening to them and understanding what medications and therapies helped them.

Once he was ready for discharge from the hospital, he was sent home with prescriptions of a seven-ay course of trimethoprim/sulfamethoxazole double strength and a higher dose of quetiapine (200 mg oral daily). He was referred to the hospital's CBT program that specializes in treatment of OCD and anxiety disorders.

## Discussion

Skin picking, also known as pathological picking, neurotic excoriation or dermatillomania is a primary psychogenic disorder that belongs to the group of obsessive compulsive and related disorders. Dermatillomania, or pathological skin picking was considered an impulse control disorder classified along with kleptomania, pyromania, trichillomata, pathological gambling and explosive disorder in the “Diagnostic and Statistical Manual of Mental Disorder” Fourth Edition, Text Revision [DSM-IV-TR] [12,13]. Dermatillomania, has since been studied and given priority in the past few years and it has now been included in DSM-5. Studies performed on patients with the skin picking disorder have supported the proposed diagnostic criteria of its inclusion [14]. From a global standpoint, the prevalence of dermatillomania is unknown; however, numerous studies have demonstrated that 2% of the patients presenting at dermatology clinics and 9% of patients with pruritus suffer from this disease. It is noteworthy to point out that studies have shown that there is a high prevalence of dermatillomania among college students (3.8%) and patients suffering from body dysmorphic disorder (28%) [12,15,16].

Dermatillomania has been associated with OCDs, body dysmorphic disorders, trichotillomania and anxiety. Patients with OCD pick at their skin to remove self-perceived dirt or contaminants, whereas patients with body dysmorphia pick their skin in order to improve slight imperfections on this skin [13]. Studies have demonstrated a female to male ratio of 7:1 in this disorder, with women being affected far more than men. The disease often manifests between 30-40 years; however, there have been reports of patients suffering from it with ages ranging from three years to 82 years. The disease can prolong for a few months to 33 years [17,18].

There are multiple triggers involved that cause skin picking or dermatillomania, including stress, anxiety, anger, boredom and even the sense of unevenness of the skin or skin discoloration. Picking of the skin starts off as an unconscious act; however, overtime it becomes conscious and deliberate. Episodes can last from a few minutes to 12 hours per day. Patients report usually using their finger nails to pick at their skin however they may use pins, tweezers, razors and other sharp objects. The skin that is removed, is either discarded and in some instances even consumed [15,17,19].

Patients describe uncontrollable pruritus before a skin picking episode; however, some patients also report skin sensations such as warmth, burning, pain and dryness. Patients usually pick on preexisting skin lesions such as acne scars, insect bites or scabs; however, some patients may also pick at normal skin. Continuous picking eventually leads to lesions in various stages of healing along with post-inflammatory hyperpigmentation and scarring. Lesions are mostly located in areas that are easily accessible by the patient such as the face or extensor surfaces of the extremities as in the case with our patient who presented with lesions on his toe and sole of the foot [12,17]. It can lead to severe life-threatening complications such as septicemia, massive blood loss by picking at skin near a major blood vessel and even intracranial infections [13,17,19].

The list of diagnosis that should be kept as differentials should include, uremia, hepatitis, xerosis, urticaria, and neoplasms, most importantly lymphomas. The differential diagnosis for the psychiatric condition should include, delusions, hypochondriasis, major depression disorder and anxiety [12].

Treatment of dermatillomania is a multipronged approach and should include the treatment of the underlying disease, treatment for pruritus, treatment of the underlying psychiatric illness and topical treatment of the lesions. SSRIs have proved to be the most effective in treating the psychiatric component of dermatillomania and SSRIs have shown immense efficacy in patients suffering from OCD [4,18]. N-acetyl cysteine (NAC) has been described in the literature as having potential benefits. Opioid antagonists have been described in case reports as being efficacious [4]. Non-pharmacological treatments such as cognitive behavioral therapy, habit reversal therapy and support groups have also proved to be helpful and are well tolerated amongst patients suffering from dermatillomania [4,18,19].

## Conclusions

Though the prevalence of the skin picking disorder or dermatillomania is unknown, it can lead to severe skin and soft damage and in extreme cases can even lead to hospitalization. Early identification and establishing the diagnosis, enrolling into a non-pharmacological treatment like cognitive behavioral therapy and habit reversal exercises could prevent further complications and permanent damage and disfigurations. 
